# High frequency of the Duffy-negative genotype and absence of *Plasmodium vivax* infections in Ghana

**DOI:** 10.1186/s12936-021-03618-0

**Published:** 2021-02-17

**Authors:** Charles A. Brown, Prince J. Pappoe-Ashong, Nancy Duah, Anita Ghansah, Harry Asmah, Edwin Afari, Kwadwo A. Koram

**Affiliations:** 1 School of Biomedical and Allied Health Sciences, College of Health Sciences, University of Ghana, Legon, Ghana; 2 Noguchi Memorial Institute for Medical Research, College of Health Sciences, University of Ghana, Legon, Ghana; 3 School of Public Health, College of Health Sciences, University of Ghana, Legon, Ghana

**Keywords:** *Plasmodium vivax*, Malaria, Duffy blood group, Duffy-negative, Ghana

## Abstract

**Background:**

Recent studies from different malaria-endemic regions including western Africa have now shown that *Plasmodium vivax* can infect red blood cells (RBCs) and cause clinical disease in Duffy-negative people, though the Duffy-negative phenotype was thought to confer complete refractoriness against blood invasion with *P. vivax*. The actual prevalence of *P. vivax* in local populations in Ghana is unknown and little information is available about the distribution of Duffy genotypes. The aim of this study was to assess the prevalence of *P. vivax* in both asymptomatic and symptomatic outpatients and the distribution of Duffy genotypes in Ghana.

**Methods:**

DNA was extracted from dried blood spots (DBS) collected from 952 subjects (845 malaria patients and 107 asymptomatic persons) from nine locations in Ghana. *Plasmodium* species identification was carried out by nested polymerase chain reaction (PCR) amplification of the small-subunit (SSU) rRNA genes. For *P. vivax* detection, a second PCR of the central region of the *Pvcsp* gene was carried out. Duffy blood group genotyping was performed by allele-specific PCR to detect the presence of the *FY*^*ES*^ allele.

**Results:**

No cases of *P. vivax* were detected in any of the samples by both PCR methods used. Majority of infections (542, 94.8%) in the malaria patient samples were due to *P. falciparum* with only 1 infection (0.0017%) due to *Plasmodium malariae,* and 2 infections (0.0034%) due to *Plasmodium ovale*. No case of mixed infection was identified. Of the samples tested for the *FY*^*ES*^ allele from all the sites, 90.5% (862/952) had the *FY*^*ES*^ allele. All positive samples were genotyped as FY*B-33/FY*B-33 (Duffy-negative homozygous) and therefore classified as Fy(a−b−).

**Conclusions:**

No cases of *P. vivax* were detected by both PCRs and majority of the subjects tested carried the *FY*^*ES*^ allele. The lack of *P. vivax* infections observed can be attributed to the high frequency of the *FY*^*ES*^ allele that silences erythroid expression of the Duffy. These results provide insights on the host susceptibility for *P. vivax* infections that had not been investigated in Ghana before.

## Background

Malaria remains the most important parasitic infection in the world, with 228 million cases in 2018 (95% confidence interval [CI] 206–258 million) [1], caused by infection with one or more of the six species of *Plasmodium* parasites. Two species, *Plasmodium falciparum* and *Plasmodium vivax*, are responsible for most of the morbidity and mortality due to malaria globally [2, 3]. However, *P. vivax* malaria does not attract as much attention in almost every aspect as does the deadlier *P. falciparum* malaria because, traditionally, the infection was thought to be benign and self-limiting [4, 5]. Recent evidence is however challenging this long-held notion of the benign nature of *P. vivax* malaria, demonstrating that infection with *P. vivax* can also result in severe illness and death [6]. Globally, 53% of the *P. vivax* burden is in the WHO South-East Asia Region and *P. vivax* is the predominant parasite (75% of malaria cases) in the WHO Region of the Americas [1]. In 2018, an estimated 704,000 (95% CI 91,000–1,813,000) *P*. *vivax* malaria cases were reported in Africa [1].

An important biological difference between *P. vivax* and *P. falciparum* is that only *P. vivax* merozoites use the Duffy (Fy) antigen receptor for chemokines (DARC) to invade erythrocytes [7, 8]. The DARC-coding gene is polymorphic with multiple alleles as the codominant FY*A and FY*B, which encode for the two antigens—Fya and Fyb. Four genotypes are possible as a result of the combination of the major alleles, Fy(a+b+), Fy(a+b−), Fy(a−b+) and Fy(a−b−) [9]. The first three correspond to a Duffy-positive phenotype, mostly prevalent in Asian and in Caucasian populations and the last one corresponds to the Duffy-negative phenotype, mainly prevalent in African people, who are consequently deemed to be refractory to *P. vivax* infection. The Fy(a−b−) genotype results from a point mutation, − 33 T → C, in the promoter region of allele FY*B, in the GATA box region [10], preventing transcription and resulting in the null ‘erythrocyte silent’ (ES) phenotype.

Until now, the Duffy-negative phenotype was viewed as giving almost total protection against infection with *P. vivax* because it prevents *P. vivax* from invading host erythrocytes and completing its complex life cycle [11]. Field observations indicate that the conclusion of the absolute dependence on the presence of Duffy on the red cell for *P. vivax* infection and development in the red cell no longer holds true because of a number of reports concerning findings of *P. vivax* in the blood of Duffy-negative persons in Brazil [12], Ethiopia [13, 14], Madagascar [15], Kenya [16], Equatorial Guinea and Angola [2] including West African countries, such as Mauritania [17], Cameroon [18, 19], Mali [20], and Benin [21]. Thus, contrary to expectation, there is evidence of *P. vivax* transmission even in areas mapped with highest Duffy-negativity frequencies [20, 22, 23].

The exact frequency of Duffy blood group is poorly documented across Africa, as indeed few populations have been surveyed and there are large gaps in the documentation on Duffy genotypes and phenotypes across Africa [24], with Ghana being no exception. Culleton et al. [25] have concluded that there are sufficient numbers of Duffy-positive individuals in some areas in Africa to maintain *P. vivax* transmission in areas where the majority of the population is Duffy-negative. The first objective of the present study was to evaluate the *P. vivax* circulation among both symptomatic and asymptomatic outpatients seeking medical care in various parts of Ghana. The second objective was to explore the Duffy antigen genotype frequency among the study population.

## Methods

### Study areas

**Fig. 1:**
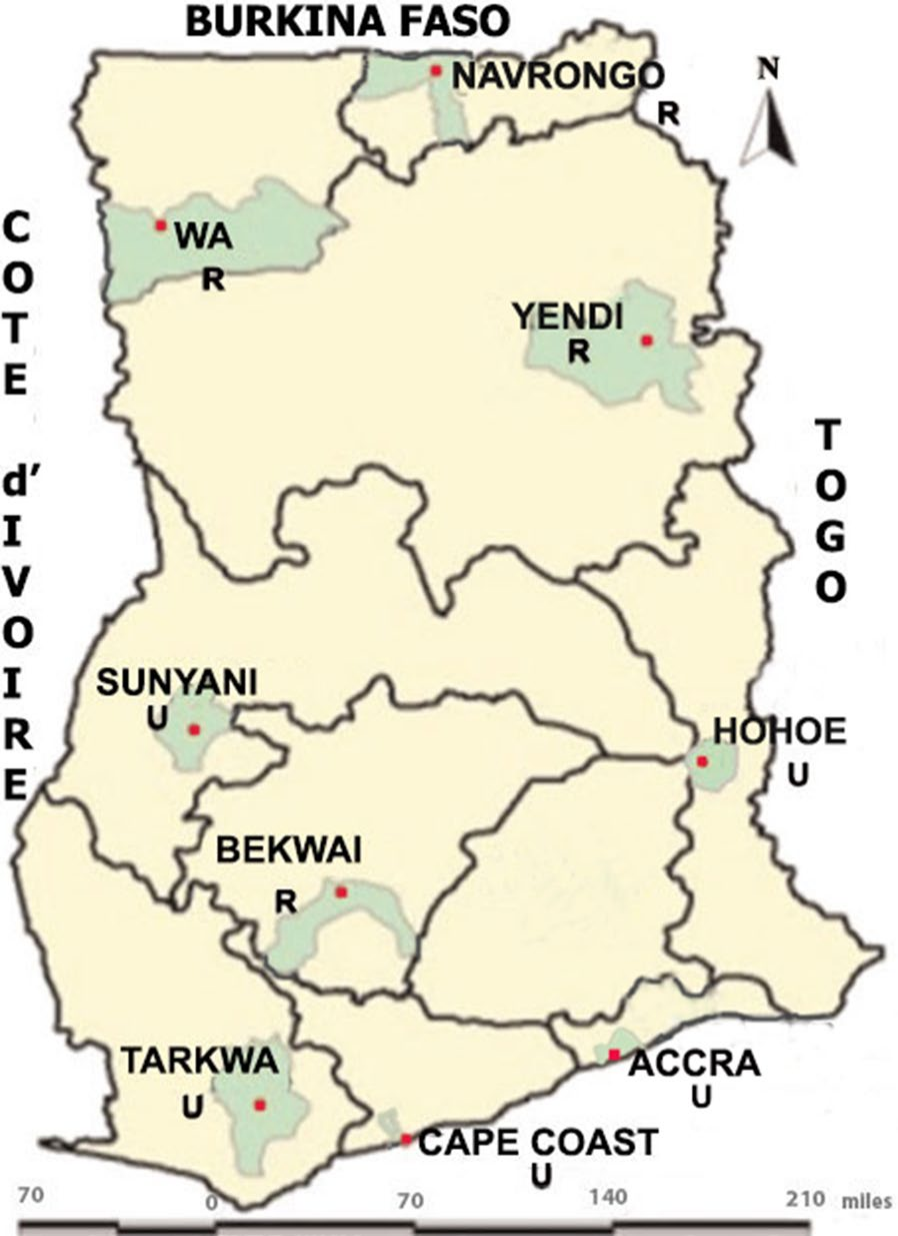
A map of Ghana showing the 9 collection sites. *U* urban, *R* rural (Adapted from Duah et al. [28])

The study was conducted in nine sites across Ghana (Fig. 1): Accra, Bekwai, Cape Coast, Hohoe, Tarkwa, Sunyani, are urban sites, and Navrongo, Wa, and Yendi are rural sites. With the exception of Accra, the other eight are sentinel sites used as part of a surveillance programme for monitoring malaria drug resistance in Ghana. The description of the sentinel sites has already been published [26–28]. The sites are located in three ecological zones of Ghana.

The Accra metropolis is the capital of Ghana and has an estimated population of 2,052,341 in 2019 [29]. The city experiences generally low and erratic rainfall pattern. Annual rainfall in the metropolis is currently 1190 mm [30]. Most of the rainfall is recorded between March and July. Malaria transmission in the metropolis is perennial.

### Sample collection and preparation

Sampling was both prospective and retrospective. Retrospective blood samples were collected by finger prick on filter paper (Whatman 3 MM filter paper) from subjects in the study areas. Informed consent was sought from all subjects. Prospective blood samples, all from Accra, were obtained from the blood banks at the Korle Bu Teaching Hospital and 37 Military Hospital to investigate the frequency of the Duffy allele. Samples were collected from 2013 to 2017.

### DNA extraction

Dried blood spots (DBS) on filter paper were cut into small pieces with scissors and transferred into 1.5-ml microtubes. For lysis, a modified salting out DNA extraction protocol with several modifications based on [31] was used. Briefly, 200 µl TNES digestion buffer (10 mM Tris–HCl (pH 7.5), 400 mM NaCl, 100 mM EDTA, 0.60% SDS) was added to the filter papers, followed by the addition of 6 µl of proteinase K (10 mg/ml) to each tube and incubation at 55 °C overnight in a heat block (Thermo Block TDB-120, Warren, USA). Samples were retrieved, 200 µl 5 M NaCl was added to each tube and vortexed briefly. The contents were then spun at 15,000 rpm for 10 min. The supernatants were transferred into 1.5 ml Eppendorf tubes, and 800 µl of cold absolute ethanol added to each tube and rocked gently back and forth. Samples were stored in a − 18 °C freezer for 3 h. They were retrieved, allowed to thaw, and spun at 14,000 rpm for 30 min. Then absolute ethanol was carefully poured off the pellet, 500 µl 70% ethanol added and the tubes spun again at 14,000 rpm for 5 min. After the spun, the supernatant was poured off and the tubes were blotted on filter paper and air-dried. The formed pellets were finally re-suspended in 200 µl TE (10 mM Tris–HCl, pH 8.0; 1 mM EDTA, pH 8.0) and stored at − 21 °C.

### Detection and identification of *Plasmodium* species

Detection of malaria infection and identification of *Plasmodium* species for all DBS samples were carried out using the nested-PCR amplification of the SSU rRNA genes as described by Snounou et al*.* [32]. For each PCR run, a negative control (sterile distilled water) and a positive control (*P. falciparum* 3D7 DNA) were used. PCR was performed using OneTaq® Quick-Load® 2X Master Mix with standard buffer from NEB (New England Biolabs Inc., Ipswich, MA, USA). PCR reactions were carried out in a SEEAMP™ SCE1000 thermal cycler (Seegene Inc., Seoul, Korea). Primers and PCR conditions are shown in Table 1.

**Table 1:** Oligonucleotide primers and PCR conditions used in this study

Purpose [Ref.]	Primer name	Sequence (5′–3′)	Amplicon (bp)	PCR conditions
*Plasmodium* sp. [32]	rPLU5	CCTGTTGTTGCCTTAAACTTC	1100	94 °C × 5 min, 35 cycles (94 °C × 60 s, 60 °C × 90 s, 68 °C × 60 s), 68 °C × 10 min
	rPLU6	TTAAAATTGTTGCAGTTAAAAC		
*P. falciparum* [32]	rFAL1	TTAAACTGGTTTGGGAAAACCAAATATATT	205	94 °C × 5 min, 35 cycles (94 °C × 60 s, 55 °C × 90 s, 68 °C × 60 s), 68 °C × 10 min
	rFAL2	ACACAATGAACTCAATCATGACTACCCGTC		
*P. vivax* [32]	rVIV1	CGCTTCTAGCTTAATCCACATAACTGATAC	120	94 °C × 5 min, 35 cycles (94 °C × 60 s, 55 °C × 90 s, 68 °C × 60 s), 68 °C × 10 min
	rVIV2	ACTTCCAAGCCGAAGCAAAGAAAGTCCTTA		
*P. malariae* [32]	rMAL1	ATAACATAGTTGTACGTTAAGAATAACCGC	144	94 °C × 5 min, 35 cycles (94 °C × 60 s, 55 °C × 90 s, 68 °C × 60 s), 68 °C × 10 min
	rMAL2	AAAATTCCCATGCATAAAAAATTATACAAA		
*P. ovale* [32]	rOVA1	ATCTCTTTTGCTATTTTTTAGTATTGGAGA	800	94 °C × 5 min, 35 cycles (94 °C × 60 s, 55 °C × 90 s, 68 °C × 60 s), 68 °C × 10 min
	rOVA2	GAAAAGGACACATTAATTGTATCCTAGTG		
*P. vivax* [33]	VivF	TCCATCCTGTTGGTGGACTT	700	94 °C × 5 min, 35 cycles (94 °C × 60 s, 60 °C × 90 s, 68 °C × 60 s), 68 °C × 10 min
	VivR	TCACAACGTTAAATATGCCAG		
Duffy genotypes [34]	GATAFY2	CTCATTAGTCCTTGGCTCTTAC	711	94 °C for 5 min, 40 cycles (94 °C × 30 s, 56 °C × 30 s, 68 °C × 60 s), 68 °C × 10 min
	FYAB2	CTCATTAGTCCTTGGCTCTTAT		
	FYAREV	AGCTGCTTCCAGGTTGGCAC		
	FYBREV	AGCTGCTTCCAGGTTGGCAT		
Human growth hormone gene [35]	HGH-F	TGCCTTCCCAACCATTCCCTTA	434	94 °C for 5 min, 40 cycles (94 °C × 30 s, 56 °C × 30 s, 68 °C × 60 s), 68 °C × 10 min
	HGH-R	CCACTCACGGATTTCTGTTGTGTTTC		

### Genotyping of *Pvcsp* genes

Further *P. vivax* parasite detection was carried out by analysis of the central region of the *Pvcsp* gene, following a slightly modified version of the protocol described by Alves et al*.* [33]. PCR was performed using OneTaq® Quick-Load® 2X Master Mix with standard buffer (NEB) and 0.4 µM of each primer. PCR reactions were carried out in a SEEAMP™ SCE1000 thermal cycler (Seegene Inc., Seoul, Korea). All PCR runs included a *P. vivax* positive control (courtesy of Michael Alifrangis). Primers and PCR conditions are shown in Table 1.

After the reaction 10 µl of the PCR product was run by electrophoresis at 120 V in 2% agarose gel (Biopioneer Co, USA) stained with 0.5 µg/ml ethidium bromide (Life Technologies Co, USA) in 1× Tris acetate EDTA (TAE) running buffer (Biopioneer Co, USA) using 2 µl of blue/orange DNA loading dye (6×) (Promega Co, USA). A 100-base pair DNA ladder (NEB) was run alongside the PCR products on the gel. The gel was photographed using UV-illumination (UVIsave gel documentation system, model GAS9200/1/2/3, version 12) and analysed.

### Duffy blood group genotyping by allele-specific PCR

Duffy genotypes were determined for all blood samples by allele-specific PCR using the protocol described by Olsson et al*.* [34], with some modifications. Four PCRs were performed on each sample to genotype FY*A, FY*B, FY*A^*ES*^ and FY*B^*ES*^ alleles. The combination of GATAFY2 and FYAREV identified the FY*A^*ES*^ allele, GATAFY2 and FYBREV identified the FY*B^*ES*^ allele, FYAB2 and FYAREV primers the FY*A allele, and FYAB2 and FYBREV primers the FY*B allele. PCR was performed using OneTaq® Quick-Load® 2× Master Mix with standard buffer (NEB) and 0.4 µM of each primer. In addition, co-amplification of the human growth hormone gene (HGH) using 0.04 µM each of the HGH-F and HGH-R primers was run as an amplification control [35]. PCR reactions were carried out in a SEEAMP™ SCE1000 thermal cycler (Seegene Inc., Seoul, Korea). Primers and PCR conditions are shown in Table 1.

## Results

### Study population

A total of 952 subjects (845 malaria patients and 107 asymptomatic persons) from 9 locations in Ghana were used for the study.

### Detection and identification of *Plasmodium* species

All the 107 asymptomatic persons were negative for *Plasmodium* species detection by nested PCR. Out of the 845 malaria patient samples, only 545 (64.5%) were found to be infected with malaria parasites following PCR diagnostic assays of the 18S rRNA gene (Table 2). As expected, majority of these infections (542, 94.8%) were due to *P. falciparum* with only 1 infection due to *Plasmodium malariae* (0.0017%) and 2 infections due to *Plasmodium ovale* (0.0034%). No mixed parasitic infections were detected. No cases of *P. vivax* were detected by PCR in the 845 patient samples tested from all the study sites.

**Table 2:** Prevalence of infection in malaria patients

Sampling sites (sample size)	Number of infected patients			
	*P. falciparum*	*P. malariae*	*P. ovale*	*P. vivax*
Bekwai (128)	103	0	0	0
Cape Coast (159)	100	0	0	0
Hohoe (73)	28	0	0	0
Navrongo (102)	90	2	0	0
Tarkwa (49)	38	0	0	0
Sunyani (18)	5	0	0	0
Wa (64)	39	0	0	0
Yendi (113)	43	0	0	0
Accra (139)	96	0	1	0
Total	542	2	1	0

### Genotyping of *Pvcsp* genes

In order to confirm the results from the PCR analyses, a different gene of *P. vivax* (*Pvcsp*) was also PCR amplified but gave the same results in that the expected 1100 bp fragment was amplified in only the positive control DNA.

### Duffy blood group genotyping by allele-specific PCR

**Fig. 2:**
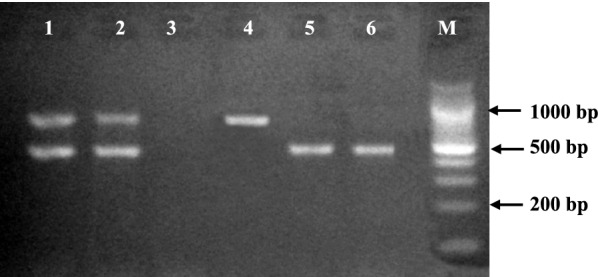
Example of an ethidium bromide-stained 1.0% agarose gel electrophoresis of allele specific PCR products for Duffy genotyping. Lanes 1 and 2 show PCR positive for both internal control and Duffy allele; Lane 3 shows a negative sample; Lane 4 shows PCR positive for only Duffy allele; Lanes 5 and 6 show PCR positive for only internal control. M = 100 bp ladder (NEB)

All the 952 subjects were Duffy genotyped by allele-specific PCR. A negative reaction was defined as the presence of only the 434 bp amplification HGH control DNA fragment. A positive reaction was defined as the presence of a clearly visible 711-bp DNA fragment with or without the amplification control band (Fig. 2). Genotyping revealed the absence of FY*B^*ES*^ allele in 90.5% (862/952) of the samples which suggested the detection of Fy(a−b−) (Table 3).

**Table 3:** Duffy blood group phenotypes and genotypes in the study population

Phenotype	Genotype	Number (frequency %)
Fy(a+b−)	FY*A/*A	0 (0)
Fy(a+b−)	FY*A/*B^*ES*^	53 (5.6)
Fy(a+b+)	FY*A/*B	15 (1.6)
Fy(a−b+)	FY*B/*B	0 (0)
Fy(a−b+)	FY*B/*B^*ES*^	22 (2.3)
Fy(a−b−)	FY*B^*ES*^/*B^*ES*^	862 (90.5)

## Discussion

Until relatively recently, *P. vivax* was rarely studied across most of sub-Saharan Africa and malaria diagnostics frequently remained limited to *P. falciparum*. Since 2010, however, evidence of the presence *P. vivax* in West Africa has emerged [21, 23, 36, 37] despite the high prevalence of Duffy-negative red blood cell phenotype. Nevertheless, no *P. vivax* infections were found in this molecular based study conducted in nine sites across Ghana.

Both symptomatic and asymptomatic outpatients were involved in this study. Malaria is hyperendemic in Ghana and 44% of outpatient visits at the various health facilities are attributed to malaria [38]. At the community level, fever or a history of fever is presumptively treated as malaria. However, according to [39], ≥ 75% of infections in malaria endemic areas are asymptomatic. This has been attributed to the development of protective immunity in adult populations against high parasitaemia and clinical disease due to the long-term continuous exposure to mosquito bites [40]. In Ghana, studies in both low and high transmission areas have found evidence of asymptomatic malaria in adult residents [41] as well as children [42] and pregnant women [43]. Asymptomatic malaria cases have been found to be higher than symptomatic cases in some studies [42, 44]. Most asymptomatic malaria infections are linked to submicroscopic parasite densities, and require the use molecular diagnostics methods [40, 45], since conventional microscopy and rapid diagnostic tests (RDTs) are of limited sensitivity.

Though *P. vivax* infections cannot be entirely ruled out in Ghana, it is important to note that an earlier study from China [46] reported a case of a 39-year-old Chinese man who had stayed in Ghana, for 6 months in 2012, for whom a microscopic examination of Giemsa-stained thin and thick blood smears initially indicated *P. vivax* infection. However, the results of a thrice conducted rapid diagnostic test were not in agreement with *P. vivax* and standard PCR analysis of the SSU rRNA gene, followed by gene sequencing, pointed to a variant *P. ovale wallikeri*. Microscopic identification of *P. ovale* and *P. vivax* due to their morphological similarities [47] may be unreliable since *P. vivax* can be misdiagnosed for *P. ovale* infections and conversely [48]. There is also potential for cross-reactivity between *P. ovale*- and *P. vivax*-specific antigens in serological screening [49].

In a 2019 case report also from China [50], a 49-year-old Chinese man was diagnosed by both microscopy and PCR as having uncomplicated *P. vivax* malaria on December 19, 2016. This was 39 days after he returned from Ghana after a stay of one and a half years. However, the Duffy genotype of the Chinese man was not given. The presence of the Fy(a−b−) phenotype outside the African continent and the Arabian Peninsula has been estimated to be at frequencies not exceeding 10% [22]. It is, therefore, highly likely that the Chinese man is Duffy-positive since the frequency of Fya among the Chinese has been estimated to exceed 97% [51].

Evidence relating to *P. vivax* transmission across Africa appears inconsistent [49]. In the West African countries were *P. vivax* infections have been recorded, Nigeria [52, 53], Mauritania [54], Mali [55], Cameroon [19], and Benin [21], the prevalence has been very low from these studies. These studies have varied in terms of sample size and diagnostic methods [56] and in some reports the Duffy antigen status of the patients was not determined [36, 52, 54]. As in this present study, extensive surveys using high-sensitivity molecular methods have repeatedly failed to diagnose *P. vivax* [25, 57].

The low prevalence of *P. vivax* infection in West Africa has been attributed to the high frequency of the Duffy-negative phenotype in this region [7, 22, 58]. In this study, 90.5% (862/952) of the malaria patients had the *FY*^*ES*^ allele and were classified as Fy(a−b−) in agreement with the report by Howes et al. [49]. It is clearly obvious that Duffy-negativity provides significant protection against *P. vivax* blood-stage infection, particularly in symptomatic patients presenting for treatment, though this protection is not absolute. This is in agreement with long-prevailing thinking that for *P. vivax* invasions to occur an interaction between the parasites and antigens of the Duffy blood group system is necessary [59, 60]. However, several other host cell receptors have recently been identified as being involved in the parasite invasion pathway of RBCs. Gruszczyk et al. [61] identified host transferrin receptor 1 (TfR1 or CD71) as an alternative receptor, critical for *P. vivax* entry into reticulocytes. CD98 has also been shown to be involved in entrance of the parasite into the host cell [62]. Lack of the Duffy antigen thus seems to places a certain restriction on the invasion mechanism, but not completely. A better understanding of the mechanisms that allow interaction between *P. vivax* and the Fya and Fyb host antigens may allow more specific assessments of the risks of *P. vivax* infection and clinical disease across the Duffy-negative populations previously considered fully protected, as well as identifying potential vaccine targets.

## Conclusions

No *P. vivax* infections were confirmed by both PCRs and the high *FY*^*ES*^ allele frequency could explain the sparse evidence of *P. vivax* infections in the samples studied across the country. Despite the fact that *P. vivax* infections cannot be entirely ruled out in Ghana, *P. vivax* malaria at present does not pose a public health risk in the country.

## Data Availability

The datasets used and/or analysed during the current study are available from the corresponding author on reasonable request.

## Abbreviations

DBS: Dried blood spots
DNA: Deoxyribonucleic acid
ES: Erythrocyte silent
PCR: Polymerase chain reaction
rRNA: Ribosomal RNA
